# Chemicals to enhance microalgal growth and accumulation of high-value bioproducts

**DOI:** 10.3389/fmicb.2015.00056

**Published:** 2015-02-17

**Authors:** Xinheng Yu, Lei Chen, Weiwen Zhang

**Affiliations:** ^1^Laboratory of Synthetic Microbiology, School of Chemical Engineering and Technology, Tianjin UniversityTianjin, China; ^2^Key Laboratory of Systems Bioengineering (Ministry of Education), Tianjin UniversityTianjin, China; ^3^SynBio Research Platform, Collaborative Innovation Center of Chemical Science and Engineering (Tianjin)Tianjin, China

**Keywords:** chemicals, microalgae, growth, accumulation, bioproducts

## Abstract

Photosynthetic microalgae have attracted significant attention as they can serve as important sources for cosmetic, food and pharmaceutical products, industrial materials and even biofuel biodiesels. However, current productivity of microalga-based processes is still very low, which has restricted their scale-up application. In addition to various efforts in strain improvement and cultivation optimization, it was proposed that the productivity of microalga-based processes can also be increased using various chemicals to trigger or enhance cell growth and accumulation of bioproducts. Herein, we summarized recent progresses in applying chemical triggers or enhancers to improve cell growth and accumulation of bioproducts in algal cultures. Based on their enhancing mechanisms, these chemicals can be classified into four categories:chemicals regulating biosynthetic pathways, chemicals inducing oxidative stress responses, phytohormones and analogs regulating multiple aspects of microalgal metabolism, and chemicals directly as metabolic precursors. Taken together, the early researches demonstrated that the use of chemical stimulants could be a very effective and economical way to improve cell growth and accumulation of high-value bioproducts in large-scale cultivation of microalgae.

## INTRODUCTION

Microalgae are autotrophic organisms, which utilize light energy, and inorganic nutrients such as CO_2_, nitrogen and phosphorus, to generate biomass and synthesize valuable metabolites. Some algal species cultivated under stress conditions accumulate specific secondary metabolites (i.e., pigments, vitamins, or lipids), which are high-value bioproducts that can be applied in the cosmetic, food, or pharmaceutical sectors (Skjanes et al., 2013). In contrast to higher plants that contain large amount of cellulose and hemicellulose, larger portion of algal biomass can be directly converted into biofuels or other high-value bioproducts *via* downstream processes (Wijffels et al., 2010; Vanthoor-Koopmans et al., 2013; Yen et al., 2013). One well-known area of such applications is microalgae-based biodiesel that has been proposed as good alternative to non-renewable fossil fuels (Sheehan et al., 1998), and another area of commercial exploitation of microalgae is the production of pharmaceutically and high-value industrial chemicals (Leu and Boussiba, 2014).

Although microalgae are known to synthesize a variety of bioproducts with potential commercial values, only a few of them, such as β-carotene and astaxanthin, have been produced at an industry-scale (Ben-Amotz, 1995; Sheehan et al., 1998; Borowitzka, 2013), which may be due to the low productivity of these products in the native microalgae and the difficulty in isolating them by economically feasible means (Clarens et al., 2010; Norsker et al., 2011; Razon and Tan, 2011; Soratana and Landis, 2011). While significant efforts have been undertaken to select high-yield strains, optimize cultivation and even modify the strains by genetic engineering in the past decades (Suen et al., 1987; Cerón Garcìa et al., 2005; Kilian et al., 2011), progress has yet to be fully satisfied.

As an alternative method to improve production or accumulation of bioproducts, chemicals as metabolic triggers or enhancers that are able to directly modulate cellular metabolism have been proposed and applied in various commercially viable microalgae. Unlike genetic modification, this approach relies on phenotypic screening and does not require specific knowledge of molecular targets in metabolic and catabolic pathways involved in synthesis of bioproducts. In a recent study, Franz et al. (2013) described a phenotypic screening of 42 chemicals for their roles on lipid metabolism in microalgae, and identified 12 chemicals that are capable of enhancing intracellular lipid levels by >100%, with three compounds (i.e., epigallocatechin gallate, CDK2 inhibitor 2 and cycloheximide) increasing intracellular lipids by 200–400% based on Nile Red fluorescence intensity. In addition, the researchers took a further step to verify these chemicals in large-scale cultures and concluded that propyl gallate and butylated hydroxyanisole could be used in large-scale applications considering the low cost of the chemicals and the lipid content increases (Franz et al., 2013), demonstrating that the application of chemical enhancer could be a valuable and practical approach in addressing the low productivity issue with microalgae-based processes. In this article, we review the recent progresses in applying chemicals to improve cell growth and accumulation of high-value bioproducts in microalgae (**Table 1**), with a focus on the molecular mechanisms of their stimulatory roles.

**Table 1:** Summary of chemicals used in enhancing growth and product accumulation in microalgae.

Species	Products	Chemicals	Reference
*Haematococcus pluvialis*	Astaxanthin	2, 4-Epibrassinolide (EBR)	Gao et al. (2013b)
*Chlorella vulgaris*	Biomass	Brassinosteroids (BRs)	Bajguz and Piotrowska-Niczyporuk (2013)
*Haematococcus pluvialis*	Astaxanthin	Jasmonic acid (JA)	Gao et al. (2012b)
*Haematococcus pluvialis*	Astaxanthin	Salicylic acid (SA)	Gao et al. (2012a)
*Haematococcus pluvialis*	Astaxanthin	Methyl jsmonate (MJ), gibberellic acid (GA_3_)	Lu et al. (2010)
*Microcystis aeruginosa*	Biomass	Polycyclic aromatic hydrocarbons	Zhu et al. (2012)
*Chlorella zofingiensis*	Astaxanthin	Pyruvate, citrate, and malic acid	Chen et al. (2009)
*Haematococcus pluvialis*	Astaxanthin	Gibberellic acid (GA_3_)	Gao et al. (2013a)
*Haematococcus pluvialis*	Astaxanthin	Salicylic acid (SA), methyl jsmonate (MJ)	Vidhyavathi et al. (2008)
*Schizochytrium* sp. HX-308	DHA	Ethanol, sodium acetate, malic acid	Ren et al. (2009)
*Chlorella vulgaris*	Biomass	Indomethacin (IM)	Piotrowska et al. (2008)
*Haematococcus pluvialis*	Astaxanthin	Fe, sodium acetate	Kobayashi et al. (1993), Choi et al. (2002), Li et al. (2008), Wang et al. (2009), Su et al. (2014)
*Synechocystis* sp. PCC680,*Anabaena*. sp PCC7120, *Scenedesmus obliquus*	Biomass, lipid, and fatty acid composition	Ethanolamine	Cheng et al. (2012)
*Haematococcus pluvialis*	Astaxanthin	Methylene blue(MB), methyl viologen (MV), H_2_O_2_, 2,2′-azo-bis(2-amidinopropane)-dihydrochloride (AAPH)	Kobayashi et al. (1993), Rioboo et al. (2011)
*Chlorococcum* sp.	Astaxanthin	H_2_O_2_, methyl viologen (MV), Fe	Ma and Chen (2001a)
*Chlorella zofingiensis*	Astaxanthin	H_2_O_2_ and NaClO	Ip and Chen (2005)
*Haematococcus pluvialis*	Carotenoid	Sodium acetate, sodium chloride, Fe, methyl viologen (MV)	Steinbrenner and Linden (2001)
*Dunaliella salina*	β-carotene	Fe, cetate, malonate	Mojaat et al. (2008)
*Chlorococcum* sp.	Free trans-astaxanthin	H_2_O_2_	Ma and Chen (2001b)
*Chlorella sorokiniana*	Biomass and lipid	2-phenylacetic acid (PAA), Indole butyric acid (IBA), 1-naphthaleneacetic acid (NAA), Gibberellic acid (GA_3_), Zeatin, thidiazuron, Humic acid, Kelp extrsct, Methanol, Fe, Putrescine, Supermidine	Hunt et al. (2010)
*Chlorella pyrenoidosa*	Biomass	Kinetin, gibberellic acid (GA_3_), indole acetic acid (IAA)	Vance (1987)
*Chlorella pyrenoidosa*	Biomass and carotenoids	Indole butyric acid (IBA), indole acetic acid (IAA), indole-3-lactic acid, tryptamine, 2-(2,4-dichlorophenoxy) acetic acid (2,4-D), naphthaleneacetic acid (NAA), N-6-benzylaminopurine, N-6-furfu-rylamineopurine, allantoin (AT)	Czerpak and Bajguz (1997)
*Chlorella vulgaris*	Biomass	Brassinosteroids (BRs)	Bajguz and Czerpak (1996)
*Chlorella vulgaris*	Biomass	Salicylic acid (SA)	Czerpak et al. (2002)
*Chlorella vulgaris*	Biomass	Diamines, polyamines	Czerpak et al. (2003)
*Monorapbidium convolutum* and *Monorapbidium minutum*	Biomass	Humic substances	Karasyova et al. (2007)
*Chlamydomonas reinhardtii and Chlorella vulgaris*	Lipid	Brefeldin A	Kim et al. (2013)
*Spirulina platensis*	Total carotenoids and α-tocopherol, glutathione (GSH), and ascorbic acid (AsA)	H_2_0_2_	Abd El-Baky et al. (2009)
*Dunaliella salina*	Biomass and glycerol	Copper	Lustigman et al. (1987)
*Haematococcus pluvialis*	Biomass and astaxanthin	Fe^2+^-EDTA, Fe^3+^-EDTA, Fe(OH)_x_^32x^, and FeC_6_H_5_O_7_	Cai et al. (2009)
*Spirulina platensis*	Biomass and free proline concentration	2-(2,4-dichlorophenoxy) acetic acid (2,4-D)	Saygideger and Deniz (2008)
*Chlorella vulgaris and Spirulina platensis*	Biomass	2, 4-Epibrassinolide (EBR)	Saygideger and Okkay (2008)
*Chlorella pyrenoidosa*	Biomass	Anthranilic acid, tryptamine, 2-phenylacetic acid (PAA), 2-(2,4-dichlorophenoxy) acetic acid (2,4-D), naphthaleneacetic acid (NAA), naphthyl-3-sulphonic acid, indole acetic acid (IAA)	Czerpak et al. (1994)
*Haematococcus pluvialis*	Carotenoids	Abscisic acid (ABA) and its analogs	Kobayashi et al. (1997, 1998)
*Selenastrum capricornutum*	Biomass	Ethyl 2-methyl acetoacetate (EMA)	Hong et al. (2008)
*Nannochloropsis salina, Nannoc hloropsis oculata, Nannochloris* sp. and *Phaeodactylum tricornutum*	Lipid	Multiple chemical triggers (Forskolin, quinacrine, butyl hydroxy anisd (BHA), epigallocatechin gallate etc.)	Franz et al. (2013)
*Chlamydomonas reinhardtii*	Biomass and fatty acid	Indole acetic acid (IAA), gibberellic acid (GA_3_), kinetin, 1-triacontanol, abscisic acid	Park et al. (2013)
*Synechocystis* PCC 6803	Biomass and lipid	Calliterpenone	Patel et al. (2013)
*Nostoc muscorum* and *Tolypothrix tenuis*	Biomass	2-phenylacetic acid (PAA)	Ahmad and Winter (1970)
*Chlorella vulgaris*	Biomass	Zeatin	Piotrowska and Czerpak (2009)
*Scenedesmus obliquus*	Biomass	Methanol	Theodoridou et al. (2002), Navakoudis et al. (2007)
*Chlorella minutissima*	Biomass	Methanol	Kotzabasis et al. (1999)
*Chlorella vulgaris*	Lipid	Fe	Liu et al. (2008)
*Dunaliella primolecta*	Biomass	Diamines and polyamines	Hourmant et al. (1994)

## PHYTOHORMONES AND ANALOGS REGULATING MULTIPLE ASPECTS OF METABOLISM

### TARGETING ON BIOSYNTHETIC PATHWAYS OF HIGH-VALUE PRODUCTS

It has been established that plants have developed a broad spectrum of molecular mechanisms to resist unfavorable environmental perturbations (Ren et al., 2009). Microalgae that share the evolutionary merits with plants also have mechanisms to deal with various environmental stress. One well-studied example is antioxidant pigment astaxanthin that plays a critical role in response to various stress conditions, such as high light, salinity, nutrient stress, and high carbon/nitrogen ratio, in chlorophyceae *Haematococcus pluvialis* (Tripathi et al., 1999; Sarada et al., 2002). The pathway of astaxanthin synthesis in *H. pluvialis* has been deciphered (Grünewald et al., 2000; Vidhyavathi et al., 2008) and several biosynthetic genes related to carotenoid have also been cloned and characterized (Lotan and Hirschberg, 1995; Sun et al., 1998; Linden, 1999; Steinbrenner and Linden, 2003; Huang et al., 2006). To increase the astaxanthin productivity, chemicals as metabolism enhancers were also evaluated recently. In one study, Lu et al. (2010) reported that gibberellic acid (GA_3_) and methyl jsmonate (MJ) played roles in regulating gene expression of *bkts* that catalyzes β-carotene to canthaxanthin in the astaxanthin biosynthetic pathway (Lu et al., 2010). More recently, Gao et al. (2012a,b, 2013a,b) found that chemicals jasmonic acid (JA), salicylic acid (SA), GA_3,_ and 2, 4-epibrassinolide (EBR) can enhance astaxanthin production to 1.458 mg/L, 2.74 mg/L, 2.39 mg/L, 2.26 mg/L, respectively; and further analysis showed that the enhancing mechanisms of chemicals were concentration-dependent. For example, the results showed that 25 mg/L JA up-regulated the transcriptional expression of *pds*, *crt*R-B, and *lyc* of the astaxanthin biosynthetic pathway (>10-fold up-regulation) the most, while 50 mg/L JA impacted the transcriptional expression of *ipi-1*, *ipi-2*, *psy*, *crt*R-B, and *crt*O than on *pds*, *lyc,* and *bkt2* more significantly (Gao et al., 2012b). Based on a correlation analysis between their maximum mRNA transcripts of five carotenoid genes and astaxanthin production, Li et al. (2010) proposed that multiple regulatory mechanisms at transcriptional, translational, and post-translational levels of astaxanthin biosynthetic genes co-existed in controlling the overall carotenogenesis process in *H. pluvialis* (Li et al., 2010). Interestingly, different modes of regulation can be issued by the same chemical in *H. pluvialis*, such as JA that up-regulated *psy*, *pds*, *crt*R-B, *lyc*, *bkt,* and *crt*O genes at the transcriptional level, and up-regulated *ipi-1* and *ipi-2* genes at both transcriptional and post-transcriptional levels, respectively; and SA up-regulated *ipi-1*, *ipi-2*, *psy*, *crt*R-B, *bkt,* and *crt*O gene at the transcriptional level, and *lyc* at the post-transcriptional level and *pds* at both levels, respectively (Gao et al., 2012a,b).

### INDUCING OXIDATIVE STRESS RESPONSES

Photosynthetic algae, like higher plants, generate reactive oxygen species (ROS) through chloroplast photosynthesis and mitochondrial respiration under stress condition, and ROS will then to be used as signal molecules to initiate production and accumulation of many bioproducts (Asada, 1994). The effects of SA and MJ on the antioxidant systems in *H. pluvialis* were investigated, and the results showed that at low concentrations, 100 μM SA increased astaxanthin content to 6.8-fold under low light (30 μmol m^-2^ s^-1^), while 10 μM MJ showed marginal increase in astaxanthin. However, at high concentration of 500 μM, both SA and MJ reduced the growth of microalgae and inhibited astaxanthin accumulation. Further mechanism analysis showed that SA at high concentrations increased superoxide dismutase activity to 4.5- and 3.3-fold and ascorbate peroxidase (APX) activity to 15.5- and 7.1-fold under low and high light, respectively, while MJ increased catalase activity (1.4-fold) under high light and APX activity (5.4-fold) under low light, suggesting the low astaxanthin accumulation may be due to the free radicals being scavenged (Raman and Ravi, 2010).

### REGULATING OTHER ASPECTS OF CELLULAR METABOLISM

Phytohormones are signal molecules synthetized by plants, and capable of efficiently regulating cellular metabolism at very low concentrations (Park et al., 2013). The application of phytohormones to improve growth and productivity has been reported, and the results with *Chlorella* species showed that use of natural and synthetic auxins, as well as their precursors, have considerable stimulating effects on algal growth and biomass composition (Czerpak et al., 1994, 1999; Czerpak and Bajguz, 1997; Hunt et al., 2010). In addition, a combination of chemicals from within the auxin family as well as with that of other families, such as 5 ppm 1-naphthaleneacetic acid (NAA) + 10 ppm GA_3_ + 1 ppm zeatin (ZT), dramatically increased biomass productivity by 170% over the control in *Chlorella sorokiniana* (Hunt et al., 2010). Another study investigated the effects of phytohormones on microalgal growth and oil accumulation for biodiesel production in *Chlamydomonas reinhardtii*. The results indicated that all five of the tested phytohormones (i.e., indole-3-acetic acid, gibberellic acid, kinetin, 1-triacontanol, and abscisic acid) promoted cell growth. In particular, hormone treatment increased biomass production by 54–69% relative to the control growth medium, demonstrating their values in decreasing cost of commercial biodiesel production (Park et al., 2013).

Brassinosteroids (BRs) are hydroxylated derivatives of 5-cholestane and important plant growth regulators in multiple developmental processes, such as cell division and cell elongation (Bajguz and Czerpak, 1996; Bajguz and Tretyn, 2003). A recent study found that BRs cooperated synergistically with auxins in stimulating cell proliferation and endogenous accumulation of proteins, chlorophylls, and monosaccharides in *C. vulgaris* (Bajguz and Piotrowska-Niczyporuk, 2013).

In terms of the molecular mechanisms, auxins and their analogs have been found to affect photosynthetic efficiency and CO_2_ fixation in microalgae. For example, a study showed that auxins had incentive effects on reactions of bonding CO_2_ to 1, 5-biphosphoribulose and photosynthetic phosphorylation. As expected, the increase in intensity of photosynthesis reactions correlated well with higher contents of chlorophylls, pheophytins, and total carotenoids in cells treated with indomethacin that shares structural similarity with natural auxins (Piotrowska et al., 2008). Other studies also indicated that low concentrations of synthetic auxins, such as 2-(2,4-dichlorophenoxy) acetic acid (2,4-D), NAA and 2-phenylacetic acid (PAA), stimulated the photosynthetic rate and chlorophylls as well as carotenoids synthesis in green algae *C. pyrenoidosa*, *Scenedesmus acuminatus,* and *S. qadricauda* (Czerpak et al., 1994, 1999, 2002; Wong, 2000).

Diamines and polyamines are polycation nitrogen compounds presented in almost all prokaryotic and eukaryotic microorganisms and belonged to specific cellular regulators of growth and metabolism (Rayle and Cleland, 1992). The study showed that in *C. vulgaris* treated with diamines and polyamines, the content of monosaccharides, primary products of Calvin cycle were intensively stimulated on 3 days of *C. vulgaris* culture, while chlorophyll content was enhanced on 9 days of *C. vulgaris* culture, indicating that the amines stimulated the dark phase of photosynthesis in the young cells, and the light synthesis phase in aging cells, respectively (Czerpak et al., 2003).

An acid growth theory has been proposed to explain the cell elongation triggered by auxins in plant cells, which refers to the auxin-induced acidification of free space in cell wall. The decrease of pH enhances the plasticity of cell wall thus contributes to the increased elongation rate of the plant tissues, and the phenomenon is presumably related to the activation of membrane-binding proton pumps by auxin (Rayle and Cleland, 1992; Hobbie et al., 1994). A study with algal *C. vulgaris* also showed that BR-stimulated cell growth depended at least partly on acid growth theory (Bajguz and Czerpak, 1996).

Cell phase and mitosis regulated by phytohormones was also reported in microalgae. A recent study showed that NAA (30 ppm) treatment stimulated higher biomass productivity between days 5 and 10 while PAA (5 ppm) treatment effected on the first 5 days in in *C. sorokiniana*, suggesting that NAA might prolong exponential phase and PAA might short initial lag phase before initiation of cell division. The combination of NAA (5 ppm) + PAA (30 ppm) showed 104% increase of biomass and demonstrated that auxins enhanced biomass growth by reducing generation time thus contributing to reducing generation time (Hunt et al., 2010). Another study on the synchronous culture of *C. pyrenoidosa* showed that the time to incipient cell division was reduced by GA and 6-furfurylaminopurine, suggesting these two phytohormones had played roles in eliminating the initial lag phase (Vance, 1987). Similarly, the cell number and dry weight of *C. vulgaris* was also significantly increased in response to optimal dose of IM (10^-7^ M) on a 5-day cultivation, suggesting that growth elicited by natural and synthetic auxins encompassed the stimulation of mitosis (Piotrowska et al., 2008).

Chlorophyll pigment presents challenges to lipid extraction and biodiesel conversion in downstream processing of algal biomass. Hence, chemicals led to higher biomass and lower pigment production will bring benefits. A study showed that the addition of NAA (30 ppm) and PAA (5 ppm) significantly increased biomass production, meanwhile decreased chlorophyll a synthesis in *C. sorokiniana* (Hunt et al., 2010). In addition, auxins at high concentrations can activate key regulatory enzyme in ethylene biosynthesis (Grossmann, 2000), and large amount of ethylene could then induce the degradation of photosynthetic pigments (Sunohara and Matsumoto, 1997).

As for other regulatory functions, an exposure of *C. vulgaris* cells to exogenous IM, synthetic analog of IAA, has been reported to increase cellular DNA level up to 48% and 20–43% more soluble proteins excreted to the environments (Piotrowska et al., 2008); and cytokinins and allantoin (AT) were found to stimulate carotenoids content by 185–190% and 124% in *C. pyrenoidosa*, possibly due to their inhibition of oxidases and dehydrogenases that are responsible for oxidation process and degradation of chlorophylls and carotenoids (Czerpak and Bajguz, 1997).

## OTHER CHEMICALS INDUCING OXIDATIVE STRESS RESPONSES

Apart from phytohormones and analogs, other chemicals capable of inducing oxidative response for enhanceing microalgal growth and accumulation of high-value bioproducts were also investigated. An early study showed that Fe^2+^, methylene blue (MB) for singlet oxygen (^1^O_2_), methyl viologen (MV) for superoxide anion radical (O_2_^-^), H_2_O_2_, and 2,2′-azo-*bis*(2-amidinopropane)-dihydrochloride (AAPH) for peroxy radical (AO_2_⋅), were capable of triggering astaxanthin biosynthesis in *H. pluvialis*, in which Fe^2+^ possibly served as an HO⋅ generator *via* an iron-catalyzed Fenton reaction (Kobayashi et al., 1993). HO⋅ or other active oxygen species (^1^O_2_, O_2_^-^, H_2_O_2_, and AO_2_⋅) might then enhance carotenoid formation in algal cyst cells by participating directly in the carotenogenic enzyme reactions as an oxidizer or an H acceptor (Beyer and Kleinig, 1989). In a recent study, Ip and Chen (2005) proposed sodium hypochlorite (NaClO) as another oxygen species to enhance astaxanthin production of *C. zofingiensis* in the heterotrophic cultivation medium.

## CHEMICALS AS METABOLIC PRECURSORS

An early study showed that an addition of 100 mM pyruvate into the culture medium of *C. zofingiensis* enhanced the yield of astaxanthin from 8.36 to 10.72 mg/L. In addition, citrate and malic acid also had the similar stimulatory effects on the formation of astaxanthin. Pyruvate might serve as a precursor for isopentenyl pyrophosphate (IPP), the carotenoid precursor in *C. zofingiensis* and *H. pluvialis*, while the stimulatory effects of citrate and malic acid on astaxanthin biosynthesis in *C. zofingiensis* could be due to their conversions to pyruvate (Chen et al., 2009). For docosahexaenoic acid (DHA) accumulation in *Schizochytrium* sp. HX-308, an addition of 4 g/L malic acid to the culture medium at the rapid lipid accumulation stage can increase DHA content of total fatty acids from 35 to 60%. In addition to functioning as a possible carbon precursor, it was speculated that malic acid added at rapid lipid accumulation stage could activate malic enzyme activity and enhance NADPH generating reaction from malic acid to pyruvate (Ren et al., 2009). In addition, ethanol was also found to enhance lipid content by 35% in *Crypthecodinium cohnii*, in which ethanol can be converted to acetyl-CoA directly and in its metabolism might generate additional reducing power NADPH for lipogenesis (Lolke et al., 2005).

**FIGURE 1:**
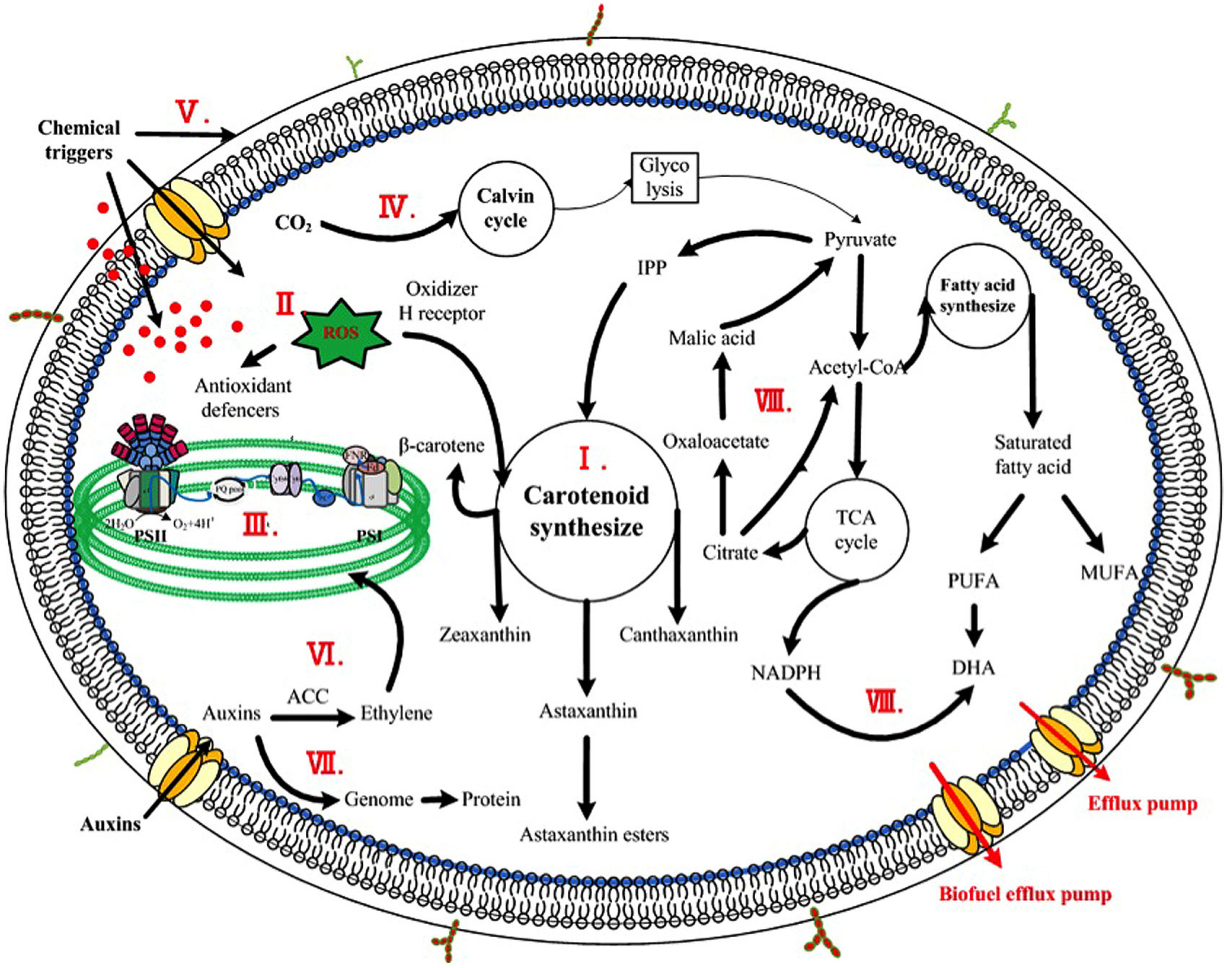
**Scheme of enhancing mechanisms of chemicals on microalgae.** The major stimulatory mechanisms were indicted inside the cell. **(I)** Chemicals targeting on biosynthetic pathways of high-value product, such as JA, SA, GA, and EBR controlling the overall carotenogenesis process in *H. pluvialis;*** (II)** Chemicals inducing oxidative stress responses, including direct or indirect addition of active oxygen species and chemical triggers inducing antioxidant production; **(III)** Phytohormones and analogs effecting on photosynthetic efficiency, namely the light phase, including photosynthetic phosphorylation, photosynthetic rate, and chlorophylls synthesis; **(IV)** Phytohormones and analogs impacting CO_2_ fixation, namely the dark phase of photosynthesis, such as diamines and polyamines stimulating production of Calvin cycle; **(V)** Phytohormones and analogs encompassed acid growth theory, alternating the plasticity of cell wall thus contributing to cell elongation; **(VI)** Degradation of photosynthetic pigments due to large amount of ethylene caused by high concentration of auxins; **(VII)** Phytohormones and analogs regulating genome and protein expression, such as IM modulating DNA and protein content in *C. vulgaris*;** (VIII)** Chemicals as metabolic precursors, such as pyruvate serving as a precursor of carotenoid synthesis thus stimulating the formation of astaxanthin and NADPH (led by malic acid) acting as a precursor of fatty acid synthesis increasing DHA content.

To aid in identifying metabolites associated with enhanced production of bioproducts, metabolomics, a measurement, and study of the small-molecule metabolites that constitute cellular metabolic networks, has been recently applied. In one study, Cheng et al. (2012) compared the metabolites between two cyanobacteria *Synechocystis* sp. PCC6803 and *Anabaena* sp. PCC 7120, and one microalga *S. obliquus* by gas chromatography coupled with time-of-flight mass spectrometry to detect important metabolites intricately tied to the lipid content in cyanobacteria and microalgae. The results showed that nine metabolites including ethanolamine were associated with the different lipid accumulation, and further study confirmed that addition of exogenous ethanolamine (2 mmol/L) could increase the lipid content by 22% in *S. obliquus* (Cheng et al., 2012). In another study, Su et al. (2014) investigated mechanism of astaxanthin induction under various stress conditions using a metabolomics and network analysis, and found that several metabolites, such as D-(+) altrose, D-ribose 5-phosphate, *L*-glutamic acid, and α-ketoglutaric acid, were positively associated with the increased astaxanthin accumulation in *H. pluvialis*. Although further confirmation is still needed, it was speculated that the increased abundances of these metabolites might contribute to the enhanced carbon flow into the astaxanthin biosynthesis (Su et al., 2014). Taken together, these early studies demonstrated that metabolomics could be a valuable tool in identifying potential metabolites for enhancing target production in algae (Zhang et al., 2010). Effective mechanisms of the chemicals were schemed in **Figure 1**.

## CONCLUSION

To produce bioproducts form microalgae in an economically feasible and sustainable way, one major hurdle that needs to be overcome is the low productivity. To address the issues, efforts have been undertaken to identify and apply chemical triggers or enhancers to enhance cell growth and accumulation of bioproducts in microalgae, and the studies have demonstrated that application of chemical triggers or enhancers could be a very practical method in large-scale fermentation of microalgae. In addition, the possible stimulatory mechanisms were also partially deciphered for some of the chemicals. However, to uncover new chemicals and expand the application, it is necessary to determine more accurately the metabolic mechanisms related to cell growth, production and accumulation of bioproducts, and the modes of action (MOA) of chemicals in microalgae. For this regard, the application of various global-focused technologies, such as genomics, proteomics, and metabolomics, could be valuable tools in the future research.

## Conflict of Interest Statement

The authors declare that the research was conducted in the absence of any commercial or financial relationships that could be construed as a potential conflict of interest.
